# Time Course Exo-Metabolomic Profiling in the Green Marine Macroalga *Ulva* (Chlorophyta) for Identification of Growth Phase-Dependent Biomarkers

**DOI:** 10.3390/md15010014

**Published:** 2017-01-10

**Authors:** Taghreed Alsufyani, Anne Weiss, Thomas Wichard

**Affiliations:** Institute for Inorganic and Analytical Chemistry, Jena School for Microbial Communication, Friedrich Schiller University Jena, Jena 07743, Germany; taghreed.alsufyani@gmail.com (T.A.); Anne.Weiss@uni-jena.de (A.W.)

**Keywords:** axenic culture, bioreactors, chemosphere, cross-kingdom cross-talk, *Maribacter*, metabolite profiling, *Roseovarius*, *Ulva*

## Abstract

The marine green macroalga *Ulva* (Chlorophyta) lives in a mutualistic symbiosis with bacteria that influence growth, development, and morphogenesis. We surveyed changes in *Ulva*’s chemosphere, which was defined as a space where organisms interact with each other via compounds, such as infochemicals, nutrients, morphogens, and defense compounds. Thereby, *Ulva mutabilis* cooperates with bacteria, in particular, *Roseovarius* sp. strain MS2 and *Maribacter* sp. strain MS6 (formerly identified as *Roseobacter* sp. strain MS2 and *Cytophaga* sp. strain MS6). Without this accompanying microbial flora, *U. mutabilis* forms only callus-like colonies. However, upon addition of the two bacteria species, in effect forming a tripartite community, morphogenesis can be completely restored. Under this strictly standardized condition, bioactive and eco-physiologically-relevant marine natural products can be discovered. Solid phase extracted waterborne metabolites were analyzed using a metabolomics platform, facilitating gas chromatography-mass spectrometry (GC-MS) and liquid chromatography-mass spectrometry (LC-MS) analysis, combined with the necessary acquisition of biological metadata. Multivariate statistics of the GC-MS and LC-MS data revealed strong differences between *Ulva*’s growth phases, as well as between the axenic *Ulva* cultures and the tripartite community. Waterborne biomarkers, including glycerol, were identified as potential indicators for algal carbon source and bacterial-algal interactions. Furthermore, it was demonstrated that *U. mutabilis* releases glycerol that can be utilized for growth by *Roseovarius* sp. MS2.

## 1. Introduction

Interactions between the marine macroalga *Ulva* and its associated bacteria impact directly on the algal morphology and physiology of both partners. Those interactions supposedly change the chemical composition of their environment, and, finally, shape the bacterial diversity of the biocoenose [1,2]. Metabolites released into the environment are considered to “act as spoken language, broadcasting signals from the genetic architecture and the environment” [3,4]. Metabolomics can help in providing a functional understanding of the physiological state of an organism or symbiotic interactions. This is particularly intriguing in the light of the exo-metabolome, which is defined by metabolites secreted into the environment by one or more organisms. Thus, the organisms form their chemosphere, interact with each other via compounds, and recruit their nutrients [5]. A non-targeted metabolomics-based approach was used to characterize the entire chemosphere of the tripartite community of *Ulva* and two associated bacteria in contrast to a traditional bioassay-guided fractionation approach that focuses on single molecules.

**Figure 1:**
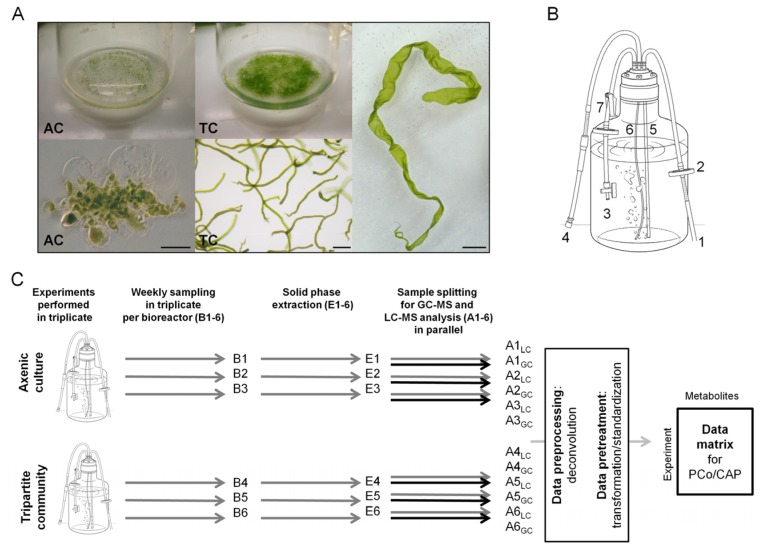
(**A**) Representative images of *Ulva mutabilis* (morphotype “slender”) are shown. Left: A five-week-old axenic culture (AC) with its aberrant morphotype and unusual cell wall protrusions (scale bar = 100 μm); center: Tripartite community (TC) of young germlings and the two bacterial strains *Roseovarius* sp. MS2 and *Maribacter* sp. MS6 with normal algal development (scale bar = 100 μm); and right: A mature specimen of *U. mutabilis* (scale bar = 1 cm); (**B**) Drawing of the 25 L bioreactor used for cultivation of *U. mutabilis*: (1) air inlet; (2) sterile HEPA-Vent (Ø = 50 mm, Whatman) filter; (3) air outlet; (4) sampling outlet; (5) bubbling tube (Duran glass, Ø = 4 mm); (6) sampling tube (Teflon, Ø 1 mm); and (7) hose clamp to control the sampling flow; (**C**) Experimental design: Three bioreactors were inoculated per treatment. Samples were taken from each bioreactor in triplicate for solid phase extraction (SPE). Each extract was then divided in half for gas chromatography-mass spectrometry (GC-MS) and ultra high performance liquid chromatography-mass spectrometry (UHPLC-MS) analyses. Three injections of each sample were used for UHPLC analysis. Upon identification of pairs of retention time and *m*/*z*, the data matrix obtained was transformed and standardized before multivariate analysis (PCoA/CAP) was conducted.

There is increasing evidence revealing cell-to-cell communication and interactions across the prokaryote–eukaryote boundary, particularly between marine bacteria and macroalgae [6]. This inter-kingdom communication is not only limited to the exchange of macronutrients, but also includes low molecular-weight infochemicals and vitamins [6]. Joint and coworkers [7] demonstrated, for example, that bacterial quorum-sensing signals, such as acyl-homoserine lactones, can serve as settlement cues for germ cells (i.e., zoids) of the marine green alga *Ulva*. These cues might direct the zoids to the respective bacterial biofilms, releasing, for instance, (additional) morphogenetic compounds (morphogens) [8]. Indeed, specific bacteria isolated from the algal surface promote growth and cell differentiation and, finally, induce algal morphogenesis via diffusible morphogenetic compounds [8]. Under axenic conditions (bacteria-free), slow-growing aberrant phenotypes, which look like calluses, have been observed in *Ulva* and particularly studied in *U. mutabilis* [8,9,10] (Figure 1A). Axenic cultures of *Ulva* have been shown to form a stable symbiotic tripartite community in the presence of the morphogenesis-inducing bacteria, *Roseobacter* sp. strain MS2 and *Cytophaga* sp. strain MS6, both isolated from *U. mutabilis* [9] (recently reclassified as *Roseovarius* sp. and *Maribacter* sp., respectively [11]). In the presence of these two bacteria, algal morphogenesis is completely recovered, mediated through bacterial morphogens [9].

*U. mutabilis* was originally collected by Føyn on the south coast of Portugal in 1952 [12,13]. From that time, the isolates, which are still in culture, have been used intensively as a convenient model for studying algal development, mainly using classic methods [14]; however, recent advances in chemical ecology and algal genetics have reinforced molecular investigations [15,16,17].

*U. mutabilis* possesses several key traits important for controlled growth and cultivation. An analysis of the vegetative cell cycle, using radioactive labeling with ^14^C-uracil, confirmed that the cell cycles are synchronous and govern circadian rhythm [18]. Additionally, blade cells of *U. mutabilis* excrete sporulation inhibitors into cell walls and the environment. These compounds, a glycoprotein and several small molecular weight compounds, are essential in maintaining the vegetative state. However, it should be noted that the gametogenesis of mature blades can be artificially induced by mincing the thallus into single mono-layered fragments and by washing-out of the sporulation inhibitors [18,19,20]. As these inhibitors can only be assessed by laborious bioassays, the identification of new growth phase biomarkers would allow regulative checkpoints in the life cycle of *Ulva* to be established. This could also prove valuable for the monitoring of aquacultures, as blade cells spontaneously differentiate into gametangia when they age [18].

In this study, the chemosphere produced by a synthetic microbiome in its most simplistic variation, the tripartite community consisting of *U. mutabilis*, *Roseovarius* sp. strain MS2, and *Maribacter* sp. strain MS6, was studied under laboratory conditions (Figure 1B). We investigated the composition of the bacterial and algal metabolites released during algal growth under standardized conditions, and compared the chemosphere between the axenic algal culture and the tripartite community using an exo-metabolomic approach (Figure 1C).

## 2. Results and Discussion

### 2.1. Experimental Design and Biological Metadata

Waterborne metabolites were isolated from the culture supernatant during *Ulva*’s growth on a weekly basis using solid phase extraction (SPE) and, subsequently, analyzed by gas chromatography (GC) and ultra-high pressure liquid chromatography (UHPLC) coupled to a time-of-flight mass spectrometer (ToF-MS). In parallel, biological metadata including (i) nutrients; (ii) sporulation- and swarming inhibitors; and (iii) algal/bacteria growth rates were determined (Figure 1). The following experimental metadata were recorded from *Ulva* cultures in bioreactors: (i) the growth of *U. mutabilis*; (ii) the depletion of nitrate and phosphate in growth media; (iii) the changes in the algal life cycle; (iv) the status of the inducibility of *Ulva*’s gametogenesis; and (v) the axenicity of the cultures, as well as the associated microbiome of the tripartite community.

#### 2.1.1. Assessment of Algal Growth and the Bacterial Community

**Figure 2:**
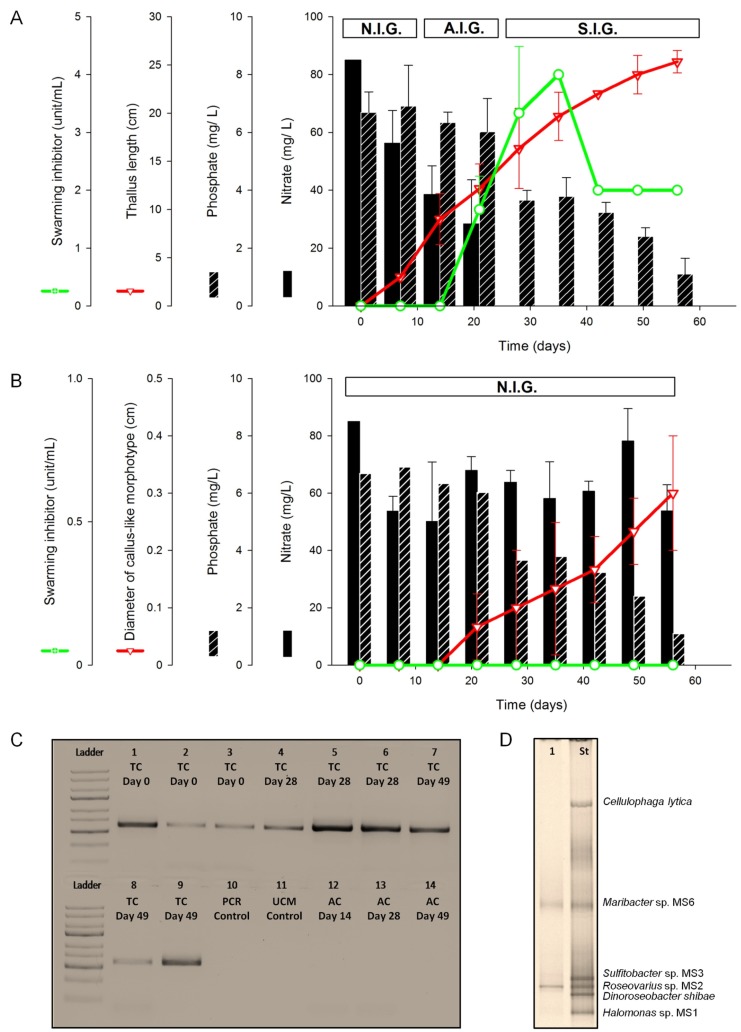
(**A**) Diagram summarizing all biotic and abiotic data (i.e., biological metadata) for *Ulva*’s tripartite community (TC), which have been collected from the onset of the algal culture (Day 0) until the mature specimen of *U. mutabilis* was ready for spontaneous gametogenesis, terminating the cultivation. The life cycle of *U. mutabilis* status was categorized according to the inducibility of the gametogenesis: non-inducible gametogenesis (N.I.G.), artificially inducible status (A.I.G.), spontaneously inducible status (S.I.G.) (mean values ± SD (*n* = 3)); (**B**) The same as (A), but *U. mutabilis* was grown under axenic conditions (AC); (**C**) Axenicity of the *Ulva* culture medium and presence of bacteria was proven by PCR of the 16S rRNA gene. An agarose gel is shown. Lanes 1–9: *U. mutabilis* inoculated with bacteria (TC) tested in three bioreactors on days 0 (1–3), 28 (4–6) and 49 (7–9); Lane 10: PCR-negative control; Lane 11: Control of *Ulva* culture medium (UCM) without *Ulva* and bacteria; Lanes 12–14: Axenic cultures (AC) tested on days 14 (12), 28 (13) and 49 (14); (**D**) Denaturing gradient gel electrophoresis analysis of PCR-amplified fragments of the bacterial 16S rRNA genes in the tripartite community on Day 49 (Lane 1) has been amplified to compare it with a mix of DNA standards derived from reference bacteria (Lane St).

*U. mutabilis* developed a normal morphology in the tripartite community, with apparent growth one week after inoculation (Figure 1A). Longitudinal growth was recorded until the end of the experiment, when the average length of a thallus was about 25 cm (Day 56 after inoculation, Figure 2A). In fact, algal biomass continuously increased, as aerification of the cultures prevented algal self-shading. As previously described [9], *U. mutabilis* did not grow properly under axenic conditions (Figure 2B) and formed dark green callus-like colonies with typical protrusions of the cell walls (Figure 1A).

Owing to the tubular and cylindrical nature of the thallus of *U. mutabilis* (slender), the length was an appropriate proxy for growth measurements. Light green thalli were observed from Day 0 to Day 14. By Day 14, they had changed to olive-green. The first brownish green and colorless thalli appeared from Day 28 onwards, indicating spontaneously induced gametogenesis.

In order to assess the bacteria within the tripartite community over time, they were routinely identified by amplifying the 16S rRNA gene using PCR (Figure 2C). It was important to use established primers, which cover a wide range of marine bacteria [21,22], in order to detect potential bacterial contaminants in the bioreactor cultures. The absence of the amplicons proved the axenicity throughout the complete period of cultivation under axenic conditions (Figure 2C). At the end of the experiment (Day 49), denaturing gradient gel electrophoresis (DGGE) analysis revealed that only the inoculated strains, MS2 and MS6, were identified in samples collected from the tripartite community (Figure 2D).

#### 2.1.2. Nutrient Depletion in *Ulva* Culture Medium (UCM)

*U. mutabilis* completed the life cycle, starting from germlings and finally forming thalli, under conditions shown in Figure 2A, i.e., when nutrient concentrations changed and nitrate was completely depleted. As the medium did not need to be renewed, the dynamic changes of the bacterial and algal exudates could be assessed during the whole algal life cycle.

Upon inoculation, the concentration of nitrate decreased within one week (*p* < 0.05). Nitrate was then continuously taken up by *Ulva* until Day 28 (Figure 2A); nitrite was not detected. The phosphate concentration decreased steadily to almost half of the initial concentration (3.5 mg/L) within the tripartite community, and remained at this level until the end of the experiment without significant changes (Figure 2A). The weekly utilization rates (UR) indicated that nitrate was consumed faster (UR_Nitrate_ = 33%) than phosphate (UR_Phosphate_ = 5%) in the tripartite community. In the fourth week post inoculation, the complete depletion of nitrate was determined (i.e., UR_Nitrate_ = 100%) and phosphate was then taken up (UR_Phosphate_ = 45%).

The only significant depletion of the nutrients in axenic cultures was recorded for nitrate (*p* < 0.05) one week post inoculation, when the initial concentration, i.e., 85 mg/L, dropped to 53 mg/L (Figure 2B) with UR_Nitrate_ = 37% (Figure 2B). Both the axenic culture and the tripartite community utilized nitrate at similar rates (33% and 37%, respectively) during the lag phase. *Ulva* is, thus, able to accumulate excess cellular nitrate for continuous growth, as was previously shown [23].

#### 2.1.3. Inducibility of Gametogenesis and Production of the Swarming Inhibitor

Spontaneous gametogenesis was not observed until four weeks, or even later, but gametogenesis could be artificially induced after two to three weeks by fragmentation of the thallus, washing, and suspension in fresh medium, which indicated the presence of sporulation and swarming inhibitors [18,19,20,24]. Discharged gametes were observed on Day 35. About 15% of the entire population had already run through the spontaneous gametogenesis by Day 42, and germlings were, hence, found one week later in all bioreactors of the tripartite community.

Previous studies have reported that the swarming inhibitor (SWI) was released into the growth medium during gametogenesis, most probably for the synchronization of gamete discharge [18,24] and can, thus, be used as a proxy for the status of *Ulva*’s life cycle. Indeed, the activity of the SWI was determined in the growth medium of the tripartite community on Day 21 (1.7 units/mL). Afterward, the activity increased significantly (*p* < 0.05) and peaked on Day 35 with 3.3 and 4 units/mL, respectively. After 35 days, the activity dropped to 2 units/mL (*p* < 0.05), and remained constant without significant change until the end of this experiment. No SWI activity could be detected in the axenic cultures (Figure 2B), as those algae do not run through gametogenesis.

Based on the inducibility of gametogenesis and the determination of the SWI activity, three phases of growth were defined in the tripartite community of *U. mutabilis*: (i) “Non-inducible gametogenesis” (N.I.G.: Day 7); (ii) “artificially inducible gametogenesis” (A.I.G.: days 14–21); and (iii) “spontaneously inducible gametogenesis” (S.I.G.: days 28–49) (Figure 2A). All axenic cultures were categorized in the phase N.I.G. (Figure 2B).

We then hypothesized that the growth stage (i.e., groups N.I.G., A.I.G., and S.I.G.) of *Ulva* at any particular time point can be revealed by the variability and composition of the metabolite profile of the chemosphere. To identify the relationships between growth stages and the chemosphere of the tripartite community, statistical analyses of the metabolite profiles using principal coordinate analysis followed by a canonical discriminant analysis with the a priori groups N.I.G., A.I.G., and S.I.G. were performed.

### 2.2. Data Analysis and Identification of Biomarkers in the Chemosphere via GC-MS

**Figure 3:**
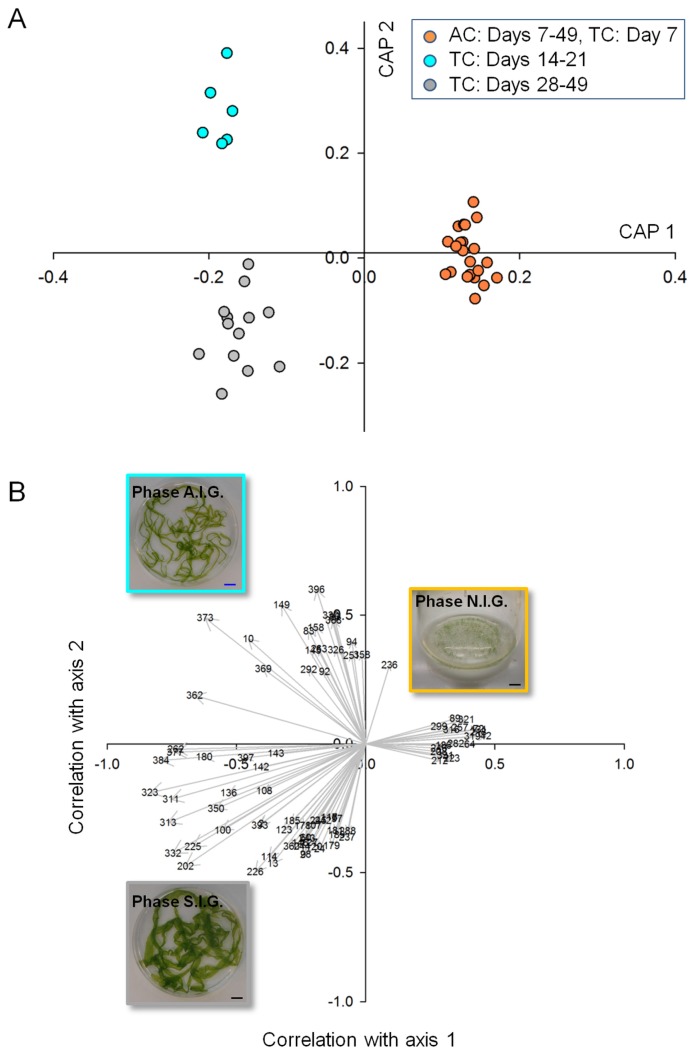
Multivariate data analysis of the exo-metabolome of *Ulva mutabilis* within the tripartite community (TC) and grown under axenic conditions (AC) after GC-MS measurements of 43 samples: (**A**) the first two canonical axes of the canonical analysis of principle coordinates (CAP) analysis are plotted. CAP analysis demonstrates the separation based on the states of gametogenesis along with the growth phases of *U. mutabilis*: non-inducible gametogenesis (N.I.G., orange), artificially inducible status (A.I.G., turquoise) and spontaneously inducible status (S.I.G., light gray); (**B**) Scaled vectors of the metabolites (ID numbers) were significant (|r| > 0.3) for the separation of the groups. The numbers refer to the metabolites in the heat map (Figure 4). Inserts show images of *Ulva*’*s* growth during the three phases, N.I.G., A.I.G., and S.I.G., selected as a priori groups for CAP analysis (bar = 1 cm).

Using the Automated Mass spectral Deconvolution and Identification System (AMDIS) and the Metabolomics Ion-based Data Extraction Algorithm (MET-IDEA), the analysis of the GC-chromatograms revealed a total of about 400 waterborne compounds detected within the tripartite community, where growth and development were observed. Canonical correlation analyses can give exact probability values and demonstrate the differences in biomarkers that reveal the changes in the exo-metabolome during *Ulva*’s life cycle, in comparison with the tripartite community and axenic growth of *Ulva* (Figure 3). Whereas differences between samples in multivariate space (PCo) were not directly apparent in the unconstrained ordination (Figure S1A), they were clearly uncovered and displayed by canonical analysis of principle coordinates (CAP) analysis (Figure 3A) using the states of gametogenesis, N.I.G., A.I.G. and S.I.G., as three a priori groups. Importantly, samples of the axenic cultures were combined over the entire experimental period (49 days), including the lag phase of the tripartite community (N.I.G.).

CAP analyses revealed that metabolite profiles significantly differed between the three growth phases (Eigenvalues: 0.9512 for axis 1 and 0.9200 for axis 2). The cross validation between the a priori groups resulted in 2% misclassification using the “leave-one-out” allocation procedure and indicates significant differences.

Correlation coefficients between each of the samples and the canonical axes were calculated by CAP. If |r| > 0.3 was chosen as a cutoff point for a correlation coefficient, which was large enough to be considered as an essential biomarker, it turned out that only the first two canonical axes had any correlation that exceeded the value in the absolute (see also Section 3).

**Figure 4:**
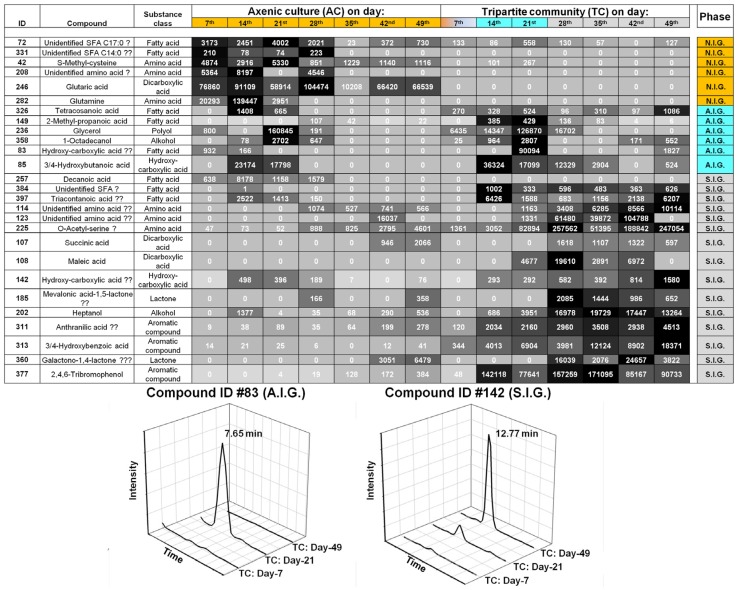
Heat map of the intensities of exo-metabolites, which were correlated with the CAP axis (|r| > 0.3) and contributed to the classification of the growth phases (N.I.G., A.I.G., and S.I.G), based on GC-MS analysis. Metabolites were extracted from *Ulva* growth medium of the tripartite community (TC) or axenic culture (AC). Relative intensities were given in the range from 0 to 250,000. Smaller values were represented by light gray and higher values by dark grey/black boxes. Metabolites marked with a “?” had reverse match between 800 and 700, and marked with “??” a reverse match of below 700. The color code refers to the three states of the gametogenesis: non-inducible gametogenesis (N.I.G., orange), artificially inducible status (A.I.G., turquoise) and spontaneously inducible status (S.I.G., light gray). Representative ion trace chromatograms of the two biomarkers, ID #83 and ID #142, for phases A.I.G. and S.I.G. over the time course, are presented.

In order to interpret which metabolites were essential for the separation, correlations with one of the two constrained canonical axes were examined (Figure 3B). The heat map, derived from the relative intensities of the 27 metabolites (including six unidentified compounds), showed that the change in their production and release correlated with the transition through the three growth phases (Figure 4). Of the 21 identified metabolites several, including glycerol, organic acids, amino acids, and 2,4,6-tribromophenol (TBP), were assessed in their putative chemical-ecological context within the *Ulva*-bacteria chemosphere (see Section 2.4).

### 2.3. Data Analysis and Identification of Important Biomarkers in the Chemosphere via LC-MS Analysis

**Figure 5:**
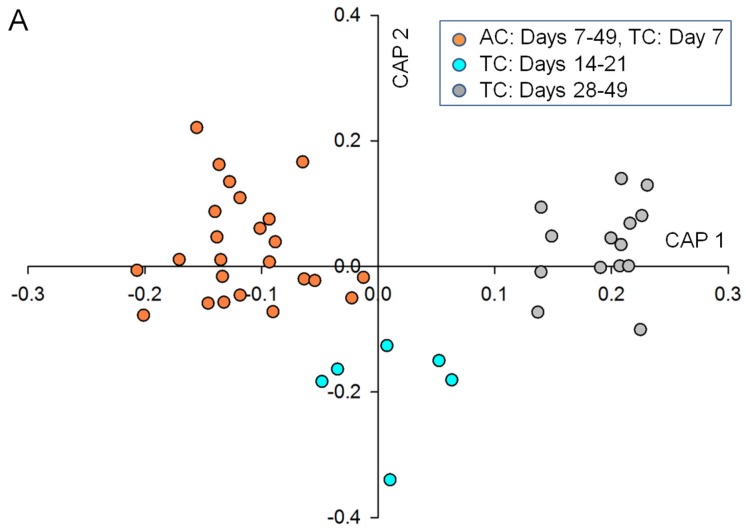

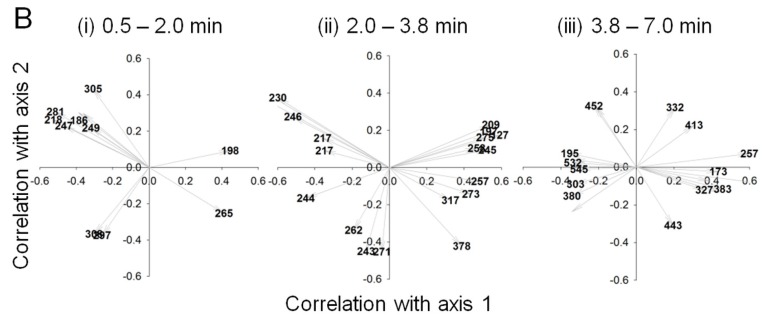
Multivariate data analysis of the exo-metabolome of *Ulva mutabilis* within the tripartite community (TC) and grown under axenic conditions (AC) as detected by UHPLC-ESI-ToF-MS. (**A**) The first two canonical axes of the CAP analysis are plotted. CAP analysis demonstrates the separation based on the states of gametogenesis along with the growth phases of *Ulva mutabilis*: Non-inducible gametogenesis (N.I.G., orange), artificially inducible status (A.I.G., turquoise) and spontaneously inducible status (S.I.G., light gray); (**B**) Scaled vectors of the metabolites (*m*/*z*) are presented significant for the separation. For better visualization, vectors of the metabolites were plotted according their retention times: (i) metabolites eluted between 0.50 and 2.00 min; (ii) 2.00 and 3.80 min; and (iii) 3.80 and 7.00 min. Here, the numbers correspond to the *m*/*z* [M+H^+^] values of the compounds, which can be also found in the heat map (Figure 6).

**Figure 6:**
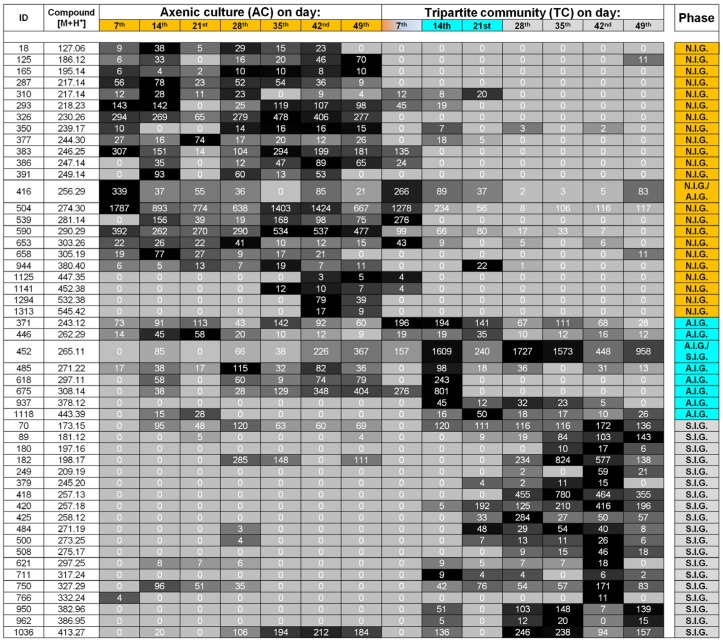
Heat map of the intensities of exo-metabolites, which were correlated with the CAP axis (|r| > 0.3) and contributed to the classification of the growth phases (N.I.G., A.I.G., and S.I.G), based on UHPLC-ESI-ToF-MS analysis. Metabolites were extracted from *Ulva* growth medium of the tripartite community (TC) or axenic culture (AC). Relative intensities were given in the range from 0 to 2000. Smaller values were represented by light gray and higher values by dark grey/black boxes. The color code refers to the three states of the gametogenesis: non-inducible gametogenesis (N.I.G., orange), artificial inducible status (A.I.G., turquoise) and spontaneously inducible status (S.I.G., light gray).

The detection of unknown metabolites is very typical for data obtained in LC-MS metabolomics [25]. Therefore, our aim was to identify those biomarkers that describe the different growth phases of the *Ulva*-bacterial tripartite community, based on its waterborne substances and to classify these as “known unknowns” [26] (Figure 5 and Figure 6). Overall, the LC-MS analyses of the exo-metabolome supported the stages categorized by GC-MS analyses. Again, whereas real group differences in multivariate space (PCo) were not apparent in the unconstrained ordination (Figure S1B), they were uncovered and displayed by the CAP (Eigenvalues: 0.9547 for axis 1 and 0.9115 for axis 2, Figure 5B), as seen in previous studies [27]. The cross validation between the a priori groups resulted in 4.5% misclassification using the “leave-one-out” allocation procedure. For better illustration, vectors of biomarkers were plotted according to their retention times (Figure 5B) indicating changes in the pattern of polarity of the waterborne metabolites. Hydrophilic waterborne metabolites (biomarkers such as #18 (*m*/*z* 127), #125 (*m*/*z* 186), #293 (*m*/*z* 218), #386 (*m*/*z* 247), #391 (*m*/*z* 249), #539 (*m*/*z* 281) and #658 (*m*/*z* 305), Figure 6) contributed significantly to the characterization of the growth phase N.I.G. and axenic culture conditions, whereas waterborne metabolites, which have been retained longer on a C18 column, due to their higher lipophilic nature, act as significant biomarkers for the growth phase S.I.G. of the tripartite community (biomarkers: #70 (*m*/*z* 173), #621 (*m*/*z* 297), #750 (*m*/*z* 327), #950 (*m*/*z* 383) and #962 (*m*/*z* 387), Figure 6). Morphogenetic compounds, such as thallusin and bacterial quorum-sensing compounds typically associated with communication between *Ulva* and bacteria [7,8], could not be detected. This is likely due to their exceedingly low concentrations; such compounds tend to be highly potent and will elicit a response even at concentrations below the MS detection limit. The heat map depicted for the first time the dynamic changes of the waterborne compounds, made possible by the fact that the growth medium did not need to be changed during the *Ulva*’s haploid stages of life cycle (i.e., from gametes as seed stock until a thallus harboring mature gametangia) (Figure 5 and Figure 6). The transition phase, A.I.G., characterized by biomarkers #371 (*m*/*z* 243), #446 (*m*/*z* 262), #485 (*m*/*z* 271), #618 (*m*/*z* 297), #675 (*m*/*z* 308), #937 (*m*/*z* 378) and #1118 (*m*/*z* 443), was of particular interest, as these correlated well with the phase when gametogenesis could be artificially induced (A.I.G.) for the first time in *Ulva*’s life cycle.

### 2.4. Biological Interpretation and Hypothesis Generation: Metabolites Shaping the Mutualistic Interaction of Ulva and Associated Bacteria

The interactions within the tripartite community depend on both the exchange of nutrients and infochemicals, which are a fundamental feature mediating the bacterial–algal interactions within the chemosphere [8]. Hereby, the experimental design of the study did not allow, in all cases, to determine whether an identified metabolite was produced by the alga or the bacteria. The following four sections discuss potential posteriori hypotheses concerning the physiological processes underlying the inter-kingdom interactions, based on chemometric and biological data analyses.

#### 2.4.1. *Ulva* Provides Glycerol as a Potential Carbon Source for Bacterial Growth

**Figure 7:**
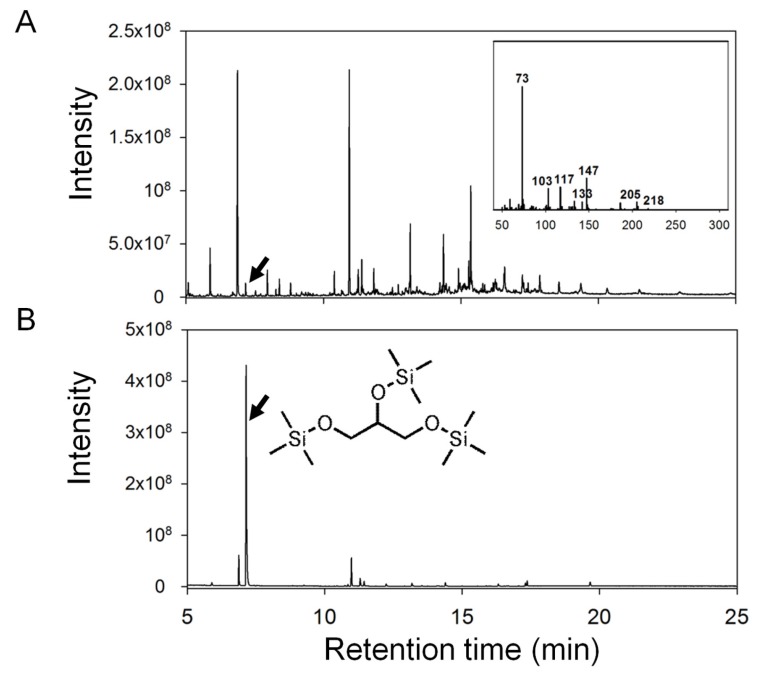

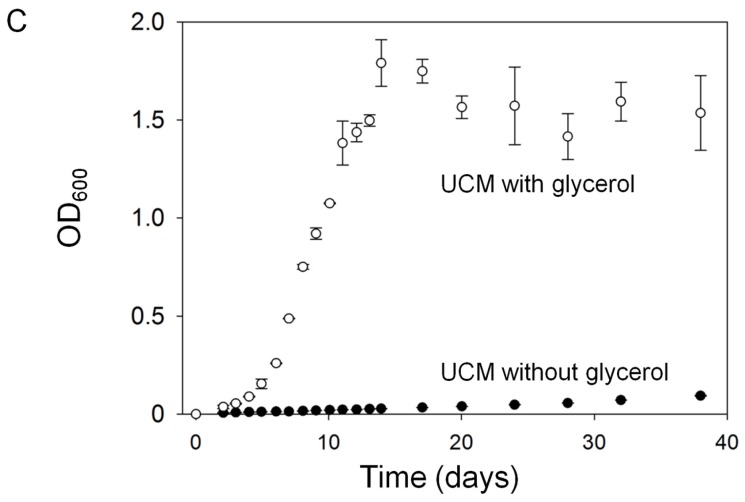
Utilization of glycerol as carbon source for *Roseovarius* sp. MS2. (**A**) Determination of glycerol in *Ulva* culture medium (UCM) of *Ulva mutabilis* under axenic conditions on Day 14 by comparison (**B**) with a reference standard upon derivatization with *N*-methyl-*N*-(trimethylsilyl)trifluoroacetamide. Total ion current chromatograms from a GC-MS analysis are presented (**A**,**B**). The mass spectrum (inset (**A**)) and the structure of the identified tris(trimethylsilyl) ether of glycerol (**B**) are shown. The molecular ion *M^+●^* is not visible. (**C**) Glycerol was tested as the sole carbon source for *Roseovarius* sp. MS2 in UCM without *Ulva*. Growth curves of *Roseovarius* sp. MS2 in UCM with (white circle) and without (black circle) supplement of 1% glycerol (*v*:*v*) are plotted (mean values ± SD (*n* = 3)).

Glycerol was found to be released by *Ulva* under axenic conditions and within the tripartite community (Figure 4 and Figure 7A,B). Therefore, growth of *Roseovarius* sp. MS2 in UCM supplemented with glycerol as a carbon source was assessed. Indeed, *Roseovarius* sp. MS2 grew well on glycerol in regard to, e.g., growth rate and final optical density (Figure 7C). Previous studies have already demonstrated that several strains of the *Roseobacter* clade harbor pathways for glycerol catabolism [28]. Glycerol released by *Ulva* likely supports growth of a wide range of bacteria resulting in biofilm formation around its holdfast.

Among these bacteria, morphogenesis-inducing bacteria would promote growth of *Ulva*, which in turn could then provide increased amounts of the carbon source and foster the bacterial–algal interactions. Additionally, several sugars, of which the identity could not be determined, were found in axenic cultures. They may work as a carbon source for *Maribacter* sp MS6, because glycerol was not utilized for growth by this strain.

#### 2.4.2. Organic Acids Might Increase the Bioavailability of Essential Trace Metals

Iron occurs primarily in hardly-soluble mineral forms in aerobic environments at alkaline pHs. However, iron can be mobilized from either a mineral or organic nutrient source by organic acids (acting as chelating agents). Higher concentrations of divalent low-molecular weight organic acids, such as maleic and succinic acids, were detected in the late growth phase and may play a role in increasing the bioavailablity of unchelated ferric iron once it becomes limited (Figure 4). It has already been demonstrated in higher plants that such mechanisms can compensate for iron deficiencies [29,30]. Certainly, further targeted screenings for organic ligands are necessary to understand the eco-physiological functions of the potential metal chelators for *Ulva* and its associated bacteria [31]. Moreover, organic acids may also buffer the medium against increasing alkalinity, or they may themselves act as nutrients [32]. With the increase in the pH of the growth medium, organic acids released by bacteria and/or the alga can act as an effective buffering system, enhancing the growth of *Ulva* by stabilizing the pH.

#### 2.4.3. Amino Acid-Mediated Signaling between *Ulva* and Bacteria

A couple of unidentified amino acids were determined in the states N.I.G. and S.I.G in addition to glutamine, *S*-methyl-cysteine and *O*-acetyl-serine (OAS) (Figure 4). The importance of amino acids for algal-bacteria interactions has already been recognized in biofilms and in plankton, exemplified by diatoms [33]. Whereas microphytobenthic assemblages take up dissolved free amino acids efficiently [34], at the same time, rapidly growing diatoms release extracellular amino acids [35]. Although the reasons for these observations could not always be deciphered, recent studies suggest, for example, the importance of the metabolic cycle of tryptophan between diatoms (*Pseudo-nitzschia multiseries*) and bacteria (*Sulfitobacter* sp.) for promoting diatom cell division via the secretion of the hormone indole-3-acetic acid (IAA) [36]. However, there is no evidence that this mode of action is also true for the interactions between *U. mutabilis*–*Roseovarius* sp. MS2 or *Sulfitobacter* sp. MS1, respectively [9]. IAA did not have an effect on *Ulva*’s growth nor on morphogenesis in experiments performed with young propagules [9,10].

#### 2.4.4. Halogenated Phenolic Substances Might Act as Deterrents in *Ulva*’s Chemosphere

The halogenated phenolic compound 2,4,6-tribromophenol (TBP) was identified in the chemosphere of the tripartite community (Figure 4). TBP is known as an antibacterial and deterrent substance against marine herbivores [37,38,39]. It inhibits the settlement of the Japanese abalone *Haliotis discus* on a brown alga [40]. Bromophenols are very common volatile compounds among red [37,41] and brown algae [42,43]. Flodin et al. [44] have already proven the existence of TBP in the green alga *U. lactuca* [44,45]. Further studies will decipher the TBP-biosynthetic pathway in *U. mutabilis* and clarify whether TBP prevents herbivory or interferes with the growth of (symbiotic) bacteria. TBP might also be released by *Ulva* in order to deter other bacteria that do not provide, for example, morphogenetic factors for *Ulva*.

### 2.5. The “Known Unknowns”

Exo-metabolomic analyses suggested the presence of several known bioactive compounds. These biomarkers, in turn, can be used as a proxy for the status of the inducibility of gametogenesis and to, generally, identify the growth phase of *U. mutabilis* when grown in the presence of the morphogenetic/bacterial factors. However, many growth-phase-dependent biomarkers, based on GC-MS and LC-MS measurements, were classified as “unknowns” due to the tremendous chemical diversity of metabolites released into the growth media and the limitations associated with the analytical platform [25,46]. Identification of each uncharacterized metabolite will be, in many ways, its own puzzle, and MS- or even bioassay-guided fractionation becomes necessary to elucidate the nature of a compound. Bacterial morphogenetic compounds might not be among them, as the biologically active concentration [9,11] seems to be far below the detection limit of the metabolomic approach applied. In fact, this is different to indol-3-acetic acid, which was recently determined to be responsible for the signaling between phytoplankton and bacteria [36]. However, the production and release of some “unknown” compounds suggest that they may be a key tool in understanding the interactions between *Ulva* and associated bacteria.

## 3. Materials and Methods

### 3.1. Cultivation of U. mutabilis and Bacteria in Bioreactors

Haploid gametophytes from the fast-growing developmental mutant “slender” (sl) of *U. mutabilis* Føyn (mating type mt+) [12] were cultured in this study (Figure 1A). A seed stock of axenic gametes was prepared for the cultivation of *Ulva* in bioreactors (Figure 1). In order to prepare the seed stock, the gametogenesis of mature gametophytes of the developmental mutant “slender” (three to four weeks old) was induced by fragmentation using a herb chopper. Fragments were washed three times with distilled water in order to remove the sporulation inhibitors (SI-1 and SI-2) [24] and were subsequently suspended in *Ulva* culture medium (UCM) and exposed to standard growth conditions in standard Petri dishes (40 mL) [24]. The gametes were discharged upon removal of the swarming inhibitor (SWI) by changing the UCM on the morning of the third day after induction. Gametes were separated from the accompanying bacteria according to a well established procedure [8] and were finally used for inoculation of an axenic (bacteria free) culture of *Ulva* in UCM. Axenicity was first tested by plating gamete samples on agar plates (marine broth +2% agar, Roth) and were subsequently further confirmed by polymerase chain reaction (PCR) of the 16S rRNA gene [9]. Axenic gametes (~6 × 10^3^ gametes) were incubated overnight in 250 mL sterile UCM in polycarbonate tissue culture flasks (BD Falcon, Franklin Lake, NJ, USA) in the dark, allowing settlement of the germ cells [5]. Those axenic germlings (feedstock) were used for the inoculation of the bioreactors (Figure 1B). Cultures were illuminated under standard conditions in a 17:7 h light/dark regime at 20 °C with 60–120 μmol photons/(m^2^·s^2^) (50% GroLux, 50% daylight fluorescent tubes; OSRAM, München, Germany). Conditions were kept constant during the entire experiment.

The bacterial strains, *Roseovarius* sp. strain MS2 (Genbank EU359909) and *Maribacter* sp. strain MS6 (Genbank EU359911), essential for *Ulva*’s growth and morphogenesis were cultivated at 20 °C in marine broth medium on an orbital shaker. These bacteria were originally isolated from *U. mutabilis* [9]. The bacterial pellets were washed three times by re-suspending them with sterile UCM before axenic germlings were inoculated with the two bacteria in order to establish the tripartite community. 

For glycerol dependent growth experiments with *Roseovarius* sp. MS2 without the algae, UCM was spiked with 1% glycerol (*v*:*v*), inoculated with the bacterium (OD = 0.001, which corresponds to the calculated OD in the UCM after inoculation) and cultivated at 28 °C.

Cultures of axenic algae and tripartite community in bioreactors (Figure 1B) were prepared under strictly sterile conditions with one-week-old axenic germlings. The final density was 5 × 10^3^ germlings/25 L. In order to set up the tripartite community, bioreactors were first inoculated with the bacteria (OD_620nm_ = 0.0001, which corresponds to the calculated OD in the TC-bioreactors after inoculation), and, subsequently, with the axenic germlings from the feedstock. Triplicates of each axenic (3 × 25 L) and the tripartite community (3 × 25 L) were conducted (Figure 1C). Duplicates of the control (2 × 10 L) were performed containing only UCM in parallel. Cultures were kept in 25 L bioreactor culture in polycarbonate bottles (Figure 1B), adapted from Reference [47].

### 3.2. Sample Collection and Storage

One liter of the supernatant was collected weekly from each bioreactor culture and filtered through a GF/C filter (glass microfiber, 1.2 μm pores , Whatman, VWR, Darmstadt, Germany) under vacuum (~500 mBar). After filtration, 1 L filtrate was extracted using EASY^®^ cartridges (SPE, Chromabond 3 mL, polar-modified polystyrene-divinylbenzene copolymer, 200 mg, Macherey-Nagel, Düren, Germany). Upon conditioning of the cartridge with 5 mL methanol, and subsequently with 5 mL distilled water, the filtrate was passed through the EASY^®^ cartridges, via Teflon tubing (flow rate = 1 L/h). After washing with 5 mL distilled water, the cartridges were air-dried and eluted by gravity with 4 mL of methanol:tetrahydrofuran (1:1, *v*:*v*) in glass vials. The vials were closed with caps fitted with PTFE-butyl-PTFE septa (VWR, Germany). An amount of 1 mL of the extract was committed to UHPLC/ESI-MS analysis and 3 mL was used for gas chromatography-mass spectrometry (GC-MS) analysis after derivatization, according to the protocol described by Vidoudez and Pohnert [47].

### 3.3. Determination of Nutrients, Biota Data and Growth

#### 3.3.1. Nitrate and Phosphate

The initial concentrations of nitrate and phosphate in the UCM were 1 mmol/L and 0.05 mmol/L, respectively, regardless of treatment. The nitrate concentration was measured by ultraviolet-visible spectroscopy (Specord M82 photospectrometer, Carl-Zeiss, Jena, Germany), based on the method of nitration of resorcinol in acidified UCM [48]. Absorption of triplicates was measured at 505 nm and concentrations determined with the molar extinction coefficient of 1.7 × 10^4^ L/(mol·cm). Phosphate was measured upon reaction with a combined reagent containing ammonium molybdate, ascorbic acid, and potassium antimonyl titrate. The resulting complex was reduced in situ to a blue colored solution. Absorption of triplicates was measured at 885 nm [49]. The concentrations of nitrate and phosphate in the UCM were determined (*C_i_*) before inoculation, and then according to the weekly sampling scheme (Figure 2).

The utilization rate of nitrate and phosphate was calculated according to the following equation

(1) $$Utilization\;Rate\;\left(100\%\right)=\frac{C_{i}-C_{a}}{C_{i}}\times \;100$$

where *C_a_* is the concentration of the nutrient following a given week, and *C_i_* is the initial concentration of the nutrient in the growth medium [50].

#### 3.3.2. Life Cycle Regulating Factors

The activity of the swarming inhibitor (SWI) was determined in the growth medium (*n* = 3), weekly, according to published protocols [18,24]. One unit of SWI is defined as the minimal amount of the factor, a compound of low molecular weight (292 Da), inhibiting gamete release in 1 mL UCM under standard conditions [18,24]. The amounts of units/mL in the growth medium are determined through a dilution series.

#### 3.3.3. Monitoring of *Ulva*’s Growth

The length of *U. mutabilis* in the tripartite communities was measured with a ruler, once, the week after inoculation, on Day 7. The average length of three thalli from each population was calculated and plotted as a function of time. The drained-weight of thalli were measured and the relative growth rate (RGR) was calculated according to References [51,52].

(2) $$GR\;\left(\%\;day^{-1}\right)=100\times ln\left(W_{2}⁄W_{1}\right)/\left(t_{2}-t_{1}\right)$$

where *W*_1_ is the drained weight (in g) at time point 1, *W*_2_ is the drained weight (in g) at time point 2, and *t*_1_ and *t*_2_ are equal to the time in days. RGR is given in % per day.

The growth in the axenic cultures was estimated by measuring the diameter of the callus-like colonies.

#### 3.3.4. DNA Extraction and PCR Amplification for Monitoring Axenicity and Bacterial Growth

PCR was performed to prove the absence or presence of bacteria in the UCM and to provide amplicons for DGGE. Samples for analysis of the tripartite community and for axenicity tests were taken weekly under sterile conditions. An amount of 50 mL of culture medium was filtered using a polycarbonate filter (Millipore ISOPORE^(TM)^, 0.2 μm GTTP 25 mm, Sigma Aldrich, München, Germany) in a polysulfone filter holder for syringes, and stored at –80 °C until extraction using a QIAamp DNA Mini Kit (Qiagen, Hilden, Germany). DNA was extracted based on the protocol of the QIAmp Blood DNA extraction kit (Qiagen Manual, 2nd Edition) metagenomics. All experiments were performed under strictly sterile conditions.

The nucleotide sequence for the forward primer, which is specific for eubacteria, contains a GC clamp (357fGC; CGC CCG CCG CGC GCG GCG GGC GGG GCG GGG GCA CGG GGG GCC TAC GGG AGG CAG CAG). The universal consensus sequence was used as reverse primer (907rM; CCG TCA ATT CMT TTG AGT TT) [53]. The PCRs, as well as the DGGE, were performed by using AmpliTag Gold DNA Polymerase (Applied Biosystems, Foster City, CA, USA) according to Grueneberg et al. [11].

### 3.4. Sample Preparation and GC-MS Analysis

#### 3.4.1. Derivatization

Five μL of a ribitol (Sigma-Aldrich, München, Germany) stock solution (4 mmol/L in water) was added to a volume of 1.5 mL of each extracted sample (Section 3.2) as internal standard. After evaporation of the solvent, 50 μL of a methoxyamine hydrochloride (Sigma-Aldrich) solution (20 mg/mL) in pyridine was added for derivatization and incubated at 60 °C for 1 h, or at 20 °C for an additional 9 h [47]. Subsequently, 50 μL of *N*-Methyl-*N*-(trimethylsilyl)trifluoroacetamide (Macherey-Nagel, Düren, Germany), supplemented with decane, pentadecane, nonadecane, octacosane, dotriacontane (each final concentration = 40 μmol/L) and hexatriacontane (final concentration = 20 μmol/L) (Sigma-Aldrich) was added to each sample for the calculation of the retention time index, and incubated at 40 °C for 1 h. The samples were transferred into 100 μL glass inserts of 1.5 mL vials and measured directly using GC-MS [47].

#### 3.4.2. GC-ToF-MS Parameters

A Waters GCT premier (Waters, Manchester, UK) orthogonal reflectron time-of-flight mass spectrometer (TOF-MS) coupled to an Agilent 6890N gas chromatograph (GC, Agilent, Santa Clara, CA, USA), equipped with a DB-5ms column (38 m long, 0.25 mm internal diameter, 0.25 μm film thicknesses, with 10 m Duraguard pre-column), was used for GC-EI-MS measurements. The samples were injected with a 7683B autosampler equipped with a 10-μL tapered, fixed needle, polytetrafluorethylene (PTFE)-tipped plunger syringe. Samples were run in a randomized order. After 20 measurements, a new deactivated glass liner (4 × 6.3 × 78.5 mm inner Ø × outer Ø × length) was used. The column and all spare parts were purchased from Agilent (Waldbronn, Germany). The GC parameters for the analysis were as follows: The carrier gas was helium at constant flow of 1 mL/min. The injection pre-dwell time was set at 0.1 min (hot needle injection). The injector temperature was 300 °C. An amount of 1 μL was injected into the split mode at a ratio of 1:5. The temperature program of the oven was set at 60 °C for 1 min at the beginning; then, the temperature was ramped up to the final temperature of 310 °C at 15 °C/min (held for 9.3 min) [5]. Calibration of the beam steering, the focusing lenses, and the dynamic range extension (DRE) was performed before every experiment. MS parameters were set as follows: Electron energy = 70 eV, trap current = 200 μA, source temperature = 300 °C, transfer line temperature = 280 °C, scan rate = 5 scans/s, mass range was set from 50 to 800 *m*/*z,* and the DRE was activated.

### 3.5. Sample Preparation and LC-MS Analysis

A Waters Acquity Ultra Performance LC (UHPLC, Waters, Eschborn, Germany) equipped with a 30 mm Fortis UHPLC C18 column (2.1 mm, 1.7 μm, Dichrom, Marl, Germany) was used for separation at 21 °C. Each sample (10 μL) was injected three times for technical replicates. The mobile phases were water spiked with 0.1% formic acid and 2% acetonitrile (A) and acetonitrile spiked with 0.1% formic acid (B) (*v*:*v*). The linear LC gradient was ramped up within 7 min from 0% to 100% B, held for 2 min at 100% B, then 9.5 min at 0% B and held for 0.5 min at 0% B. The flow rate was 0.6 mL/min [5]. The UHPLC was coupled to a quadrupole (Q)-TOF Micromass spectrometer (Manchester, UK) using an electrospray ionization source in positive ion mode with a scan rate of 1 scan/s, an inter-scan delay of 0.1 s and a scan range from 100 to 1000 *m*/*z* [54,55].

### 3.6. Data Processing

#### 3.6.1. GC-MS

All chromatograms were corrected with the Component Detection Algorithm implemented in Masslynx™ (version 4.1, Waters, Manchester, UK) for background signals. The MCQ index was set at 0.8 and the smoothing window was 3 points. The chromatograms were transferred to network common data files by the Masslynx™ DataBridge (Micromass, V4.1). Co-inverted spectra were treated in batch jobs in the Automated Mass spectral Deconvolution and Identification System (AMDIS; version 2.65, National Institute of Standards and Technology (NIST, Gaithersburg, MD, USA)). The following parameters were used: minimum match factor = 30, type of analysis = simple, component width = 32, omitted *m*/*z* values: 147, 176, 193, 207 and 219, adjacent peak subtraction = 2, resolution = low, sensitivity = medium, and shape requirement = low. The common data files and the corresponding AMDIS files were fed into METabolomics-Ion-based Data Extraction Algorithm (MET-IDEA) (version 2.03). Following parameters were applied: average peak width = 0.08, AMDIS transfer = 0.5, maximum peak width = 2, peak start/stop slope = 1.5, adjusted retention time accuracy = 0.25, peak overload factor = 0.9, MS type = TOF, mass accuracy = 0.1, mass range = 0.3, excluded ions = 73, 147, 193, 281, 341 and 415, lower mass limit = 100, and ions per component = 1 [5]. Signals corresponding to the retention index standards and ribitol were deleted prior to the chemometric analyses.

#### 3.6.2. LC-MS

MarkerLynx™ (Version 4.1, Waters, UK) was used for the acquisition of the mass-retention time pairs. This application integrates peaks using ApexTrac^TM^ peak detection and implements basic peak detection and baseline determination algorithms. The processing parameters that control peak detection and baseline placement are independent of each other and were set at a peak width of 5%, peak width of 5% height (s) = 15, peak-to-peak baseline ratio = 0, noise elimination = 0.10, intensity threshold = 10, *m*/*z* window = 0.05 amu, and at a retention time window = 1 min.

### 3.7. Statistical Analysis

Post hoc comparisons for all metadata were performed using Tukey’s HSD (honestly significant difference) test at a probability level of α = 0.05 to determine pairwise differences between treatments (Minitab 16.2.4, Friedrichsdorf, Germany).

Canonical analysis of principle coordinates (CAP) was used for multivariate data analysis to visualize differences in exo-metabolite assemblages during algal growth. The procedure couples principal coordinate analysis (PCoA), an ordination procedure and a generalization of a principal component analysis, with a canonical analysis. The unconstrained ordinates share the common feature that the experimental units are not divided into groups and they do not provide any null hypothesis that can be tested statistically. Therefore, in addition to PCoA, the CAP computer program (version 12) performs two further distinct statistical procedures: The first step is the canonical discriminant analysis, in which the experimental units are arranged in predefined groups (*a priori* hypotheses) and the null hypothesis is that the multidimensional vectors of means do not differ [56]. Then, the permutation test provided an exact, nonparametric *p*-value for that null hypothesis [57]. Within this routine, the principal coordinates (PCo) were calculated from the Bray-Curtis resemblance matrix, and potential over-parameterization was prevented by choosing the number of PCo axes (*m*) that maximized a leave-one-out allocation success to the groups [27,58,59]. Data were transformed to square root and standardized by row (i.e., samples) sums.

The canonical test statistics were significant for both GC-MS and LC-MS data sets. Using a total of 999 permutations, the *p-*value was 0.001, obtained from the permutation test using CAP [27,58]. As 0.001 is equal to 1/(999 + 1), it has been demonstrated that no randomly permuted data set had a more drastically different biomarker assemblage than that of the data set examined.

The resulting first two canonical axes and sample coordinates were then imported into SigmaPlot (version 11.0, Systat Software, Erkrath, Germany) for graphical illustration. Chemical biomarkers responsible for differences among the treatments and growth phases were identified by the strength of their correlation of the canonical discriminant axes coordinates with the indicative compounds. Depiction of the vectors of these metabolites on the CAP axis allowed determination, in which grouping was important. The Pearson correlation coefficients were scaled to the CAP range of the coordinates (usually between |r| > 0.3–0.5) to generate the vectors corresponding to each significant compound (Figure 3B and Figure 5B). Hereby, the two-tailed probability value, *p*, is equal to 0.05 (maximum *p* value applied in this study) when a Pearson correlation value of |r| = 0.3 and a sample size of *n* = 43 were given (exemplified for the data set of the GC-MS analysis). Therefore, only biomarker correlations of |r| > 0.3 were considered as significant.

### 3.8. Annotation of Metabolites Acquired by GC-MS Analysis

The spectrum of each peak retained for analysis was manually examined and identification was attempted using the software MS search (version 2.0 d, NIST). The following libraries were used: NIST, Golm Metabolome Database (Version: 121_VAR5_ALK_MSP) and MPI of Molecular Plant Physiology (Version: Q_MSRI_ID2004-03-01). A structure was accepted if the reverse match was higher than 800 and if the retention index was close to the index provided in the libraries [5]. Structures with a reverse match between 800 and 700 were tagged with a “?” and structures with a reverse match below 700 with “??”. Reverse match factors below 600 were not accepted. Finally, proposed compounds were compared with reference standards in certain cases, as illustrated for glycerol (Figure 7).

## 4. Conclusions

We analyzed the exo-metabolome during macroalgal growth in the presence of a designed and simplified microbiome. The possibility of controlling growth conditions precisely and obtaining large amounts of homogeneous sample material was a necessity to study complex exo-metabolic changes with a high temporal resolution, as has already been shown for microalgae, such as *Chlamydomonas* [60].

This study has demonstrated that the release and persistence of extracellular organic compounds depends on the interaction of *Ulva* with the tested bacteria, and on the growth stage. Differences in biomarker assemblages revealed changes in *Ulva* growth phases. Overall, metabolomics is a key tool for bridging the gap between the environment and *Ulva*’s holobiont, interacting directly via the exchange of, for example, infochemicals, and indirectly through the recruitment of, for example, nutrients. However, the majority of compounds were classified as “known unknowns”.

The GC-MS-based metabolomic data set of identified compounds, along with the metadata, leads to new hypotheses regarding the chemical ecology of *U. mutabilis*. We believe it is worthwhile pursuing more work on the structure elucidation of the phase-dependent compounds identified, because they can be used as robust proxies of the physiological status of a tripartite community. Bacteria, grown on ^13^C-labeled carbon sources, which are utilized for the synthesis of bacterial metabolites, will help to differentiate between algal and bacterial compounds in the chemosphere. In addition, the combined approach of transcriptomics and metabolomics may reveal novel insights into how the physiology of *Ulva* is changing in its time course-dependent chemosphere.

## Supplementary Materials

The following is available online at www.mdpi.com/1660-3397/15/1/14/s1: Figure S1: Multivariate data analysis (PCoA) of the exo-metabolome of *Ulva mutabilis* within the tripartite community (TC) and grown under axenic conditions (AC) after GC-MS and LC-MS analyses.

## Abbreviations

The following abbreviations are used in this manuscript:

AC	axenic culture
A.I.G.	artificially inducible gametogenesis
CAP	canonical analysis of principle coordinates
DGGE	denaturing gradient gel electrophoresis
DRE	dynamic range extension
EI	electron impact
ESI	electrospray ionization
GC	gas chromatography
IAA	indole-3-acetic acid
LC	liquid chromatography
MS	mass spectrometry
N.I.G.	non-inducible gametogenesis
OD	optical density
PCoA	principal coordinate analysis
PCR	poly chain reaction
SI	sporulation inhibitor
S.I.G.	spontaneously inducible gametogenesis
SFA SWI	Saturated fatty acids swarming inhibitor
TBP	2,4,6-tribromophenol
TC	tripartite community
ToF	time-of-flight mass spectrometer
UCM	*Ulva* culture medium
UHPLC	ultra-high pressure liquid chromatography
UR	utilization rate
