# Supplementary material for: Time Course Exo-Metabolomic Profiling in the Green Marine Macroalga Ulva (Chlorophyta) for Identification of Growth Phase-Dependent Biomarkers
Source: Mar Drugs. 2017 Jan 10;15(1):14. doi: 10.3390/md15010014 (PMC5295234; doi:10.3390/md15010014)
Supplement: Supplementary file 1 [file marinedrugs-15-00014-s001.docx]

Supplementary Materials: Time Course
Exo-Metabolomic Profiling in the Green Marine Macroalga *Ulva* (Chlorophyta) for Identification of Growth Phase-Dependent Biomarkers

Taghreed Alsufyani, Anne Weiss and Thomas Wichard


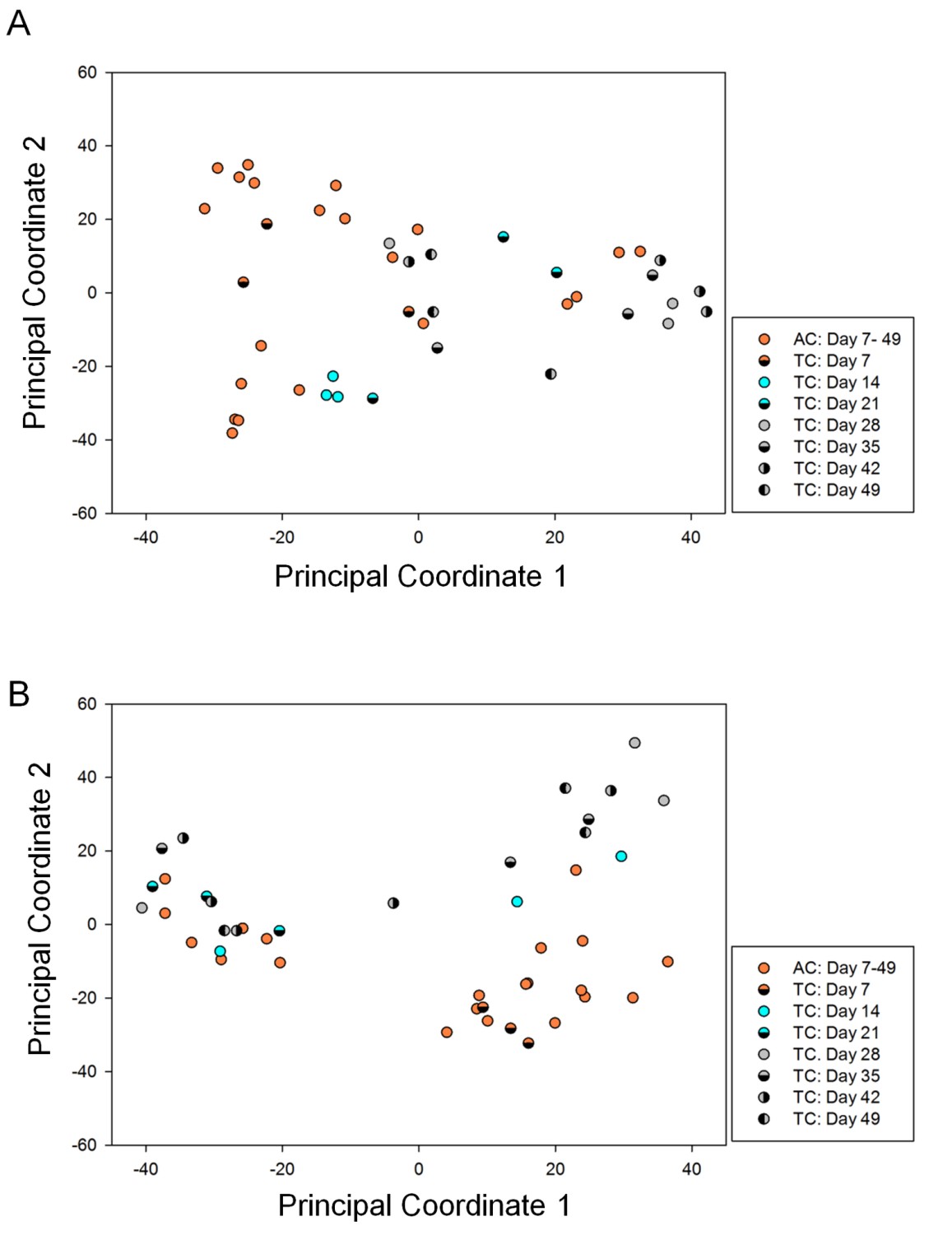


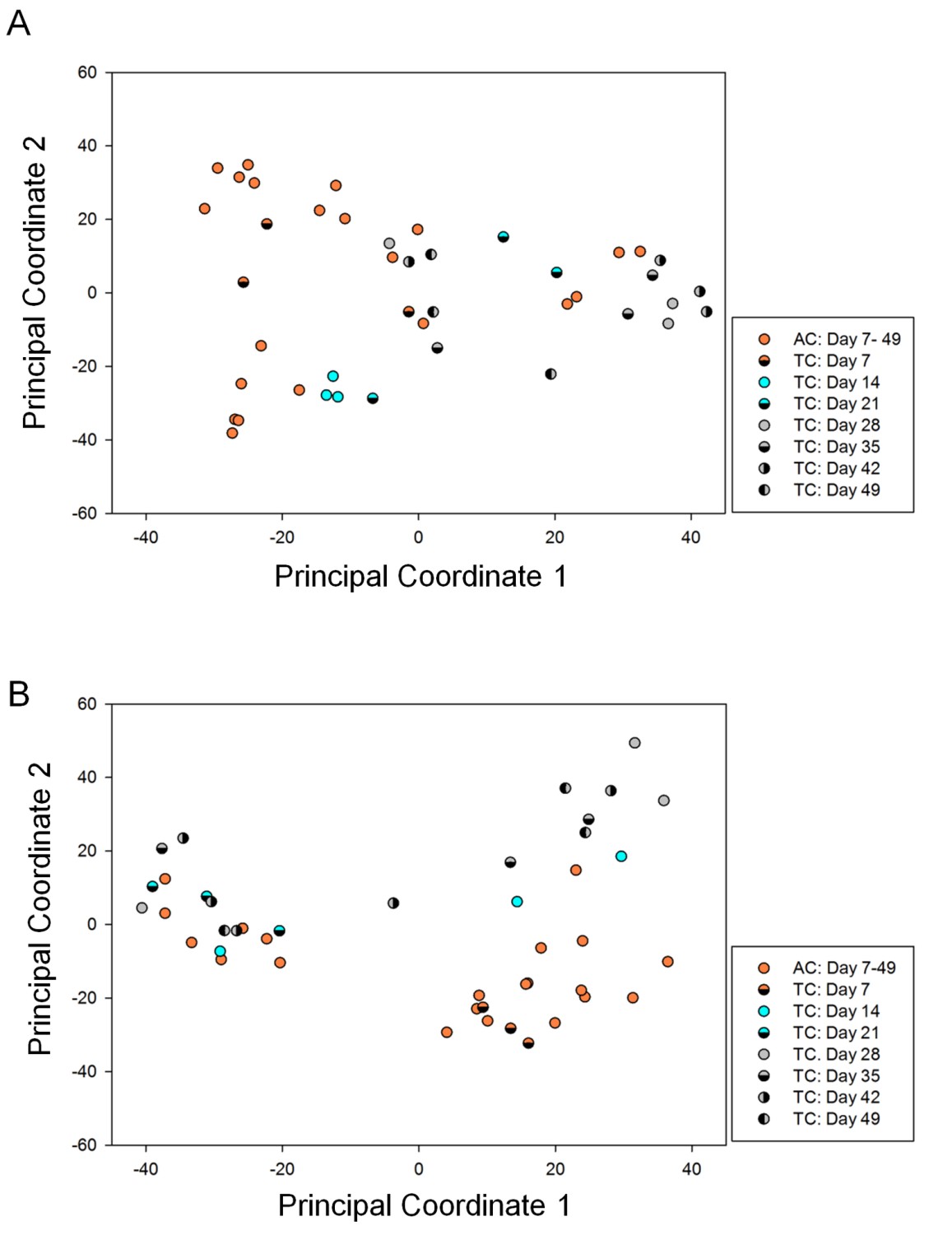


**Figure S1.** Multivariate data analysis of the exo-metabolome of *Ulva mutabilis* within the tripartite community (TC) and grown under axenic conditions (AC) after GC-MS and LC-MS. Multivariate data analysis (PCoA) of the exo-metabolome of *Ulva mutabilis* within the tripartite community (TC) and grown under axenic conditions (AC) after GC-MS (**A**) and LC-MS (**B**) measurements of 43 samples: The color code refers to the three states of the gametogenesis: non-inducible gametogenesis (N.I.G., orange), artificial inducible status (A.I.G., turquoise) and spontaneously inducible status (S.I.G., light gray). (**A**) Score plot of the first two principal coordinates of the PCoA analysis. It explained 34% variance, of which PCo-1 explained 20% and PCo-2, 14%. (**B**) Score plot of the first two principal coordinates of the PCoA analysis. It explained 33% variance, of which PCo-1 explained 20% and PCo-2, 13%, is shown.
